# EFFECTS OF HEARING INTERVENTION ON BRAIN HEALTH: FINDINGS OF THE ACHIEVE TRIAL AND AND ACHIEVE-MRI SUBSTUDY

**DOI:** 10.1093/geroni/igae098.0052

**Published:** 2024-12-31

**Authors:** Alison Huang, Frank Lin, Caterina Rosano

**Affiliations:** Chair; Co-Chair; Discussant

## Abstract

This session will present the primary findings of the ACHIEVE-MRI (Aging and Cognitive Health Evaluation in Elders Magnetic Resonance Imaging) study, a substudy of the ACHIEVE randomized controlled trial (Clinicaltrials.gov Identifier: NCT03243422). Prior observational studies have linked hearing loss with changes in brain structure and function. Hearing loss may directly modify brain morphology and exacerbate mechanistic pathways such as greater cognitive load (diversion of cognitive resources to effortful listening to the detriment of other cognitive functions) and reduced social connection. Whether treatment of hearing loss can effectively reduce age-related brain atrophy is unknown and has yet to be tested in randomized trials. The ACHIEVE trial (2018-2022) tested the effect of a best-practices hearing intervention versus health education control on three-year change in cognition (primary outcome) among 977 adults aged 70-84 years with untreated hearing loss and without substantial cognitive impairment. MRI imaging data was gathered on a subset of participants (n=445) in the ACHIEVE-MRI substudy. This session will first describe the overall study design of the ACHIEVE study and ACHIEVE-MRI substudy and then present the primary findings of the ACHIEVE- MRI substudy – the effect of the hearing intervention vs. health education control on 3-year change in brain structure and function. This session will also examine the effect of hearing intervention on speech understanding in the presence of noise. The session will close with an investigation of for whom hearing intervention may be most effective for, examining moderation by baseline levels of dementia risk in the ACHIEVE study.

